# Surgical Management of Appendicular Skeletal Metastases in Thyroid Carcinoma

**DOI:** 10.1155/2012/417086

**Published:** 2012-12-12

**Authors:** Robert L. Satcher, Patrick Lin, Nursat Harun, Lei Feng, Bryan S. Moon, Valerae O. Lewis

**Affiliations:** ^1^Department of Orthopaedic Oncology, The University of Texas MD Anderson Cancer Center, 1400 Pressler Street, FCT10.5044, Unit 1448, Houston, TX 77030, USA; ^2^Department of Biostatistics, The University of Texas MD Anderson Cancer Center, Houston, TX 77030, USA

## Abstract

*Background*. Bone is a frequent site of metastasis from thyroid carcinoma, but prognostic factors for patients who have surgery for thyroid carcinoma bone metastases are poorly understood. *Methods*. A retrospective review at a single institution identified 41 patients that underwent surgery in the appendicular skeleton for thyroid carcinoma bone metastasis from 1988 to 2011. *Results*. Overall patient survival probability by Kaplan-Meier analysis after surgery for bone metastasis was 72% at 1 year, 29% at 5 years, and 20% at 8 years. Patients who had their tumor excised (*P* = 0.001) or presented with solitary bone involvement had a lower risk of death following surgery adjusting for age and gender. Disease progression at the surgery site occurred more frequently with a histological diagnosis of follicular carcinoma compared with other subtypes (*P* = 0.023). Multivariate analysis showed that tumor subtype, chemotherapy, and preoperative radiation treatment had no effect on survival after surgery. Patients treated with radioactive iodine had better survival following thyroidectomy, but not following surgery for bone metastases. *Conclusions*. For patients undergoing surgery for thyroid cancer bone metastasis, resection of the bone metastasis, if possible, has a survival benefit.

## 1. Introduction

Distant metastases are found at diagnosis or followup in 10%–15% of patients with differentiated thyroid cancer [1–4]. Bone is the second most common site of metastasis after lung [1, 2, 4]. Patients with bone metastases (BMs), whether isolated or associated with lung metastases, have a poor prognosis, with ten-year survival rates ranging from 13%–21% [1, 5]. Several studies have examined the prognostic factors and treatment outcomes for bone metastases from thyroid cancer, but few have evaluated the effects of surgical management [2, 6–8]. 

Many of the lesions in the appendicular skeleton can be effectively managed by external beam radiation therapy (EBRT) or radioactive iodine [7, 9–12]. Some, however, require surgical intervention due to their symptoms and fracture risk [6–8]. Patients with BMs from thyroid carcinoma generally have better survival than some other carcinomas that frequently metastasize to bone, such as lung and renal cell carcinoma [3–5, 7, 13–15]. After synchronous or metachronous BMs from thyroid carcinoma, 5-year survival has been reported to be from 29% to 69% [2, 5, 7, 9, 13–15]. 

Complete resection of the BM is thought to be associated with better overall survival and can be curative [1, 7, 9, 12, 15]. Prognosis also may improve if BMs are detected earlier [4, 5, 13, 16]. A recent study showed that BMs detected by ^131^I scanning had excellent response to ^131^I therapy [5]. Repeated ^131^I therapy can be effective in targeting not only visible metastases, but also those too small to be imaged [17]. In contrast, there are no effective therapies for patients with poorly disseminated carcinomas that progress despite ^131^I therapy [5, 13, 18, 19]. Some recent studies have shown that patients with both lung metastases and BMs have similar survival as those with BMs alone, in contrast to historical beliefs [1, 20]. Their survival is better than patients with metastases to other organs, such as the liver [1, 9].

The options for limb salvage reconstructions following partial or complete bone resections have increased in the recent years [21–26]. Surgical guidelines continue to evolve and are not standardized. The goal of surgical management has continued to be to establish a durable construct that lasts the lifetime of the individual and simultaneously optimizes the quality of life via pain relief, mechanical stability, and preservation of mobility [25, 27–29]. Because there have been few studies that evaluate the outcome of the surgical management of BMs, the criteria for deciding whether these patients are treated with partial or complete BM resection remain subjective [6, 8, 15].

The goal of the present study was to determine the length of survival and prognostic factors for patients with thyroid carcinoma BMs who had surgery, as well as the type and the survival of the various means of surgical reconstruction. In particular, we wished to evaluate whether the surgical implants lasted for the life of the patient, without the need for revision. We also examined the specific impact of BM resection and histologic subtype of thyroid carcinoma on survival. 

## 2. Materials and Methods

### 2.1. Study Design

We performed a retrospective review of patients treated surgically for osseous lesions secondary to metastatic thyroid carcinoma at a single institution between 1988 and 2011. Patients were identified by interrogating the Department of Orthopaedic Oncology Surgical Database. The study was performed with the approval and in accordance with guidelines by the institutional review board. Medical records, operative reports, radiographic studies, and pathology reports were reviewed.

### 2.2. Patients

There were 8681 patients with thyroid cancer identified from institutional records. Of these, 550 developed bone metastases and 43 required skeletal surgery. The study population was composed of 22 females and 21 males. The median age at thyroid cancer diagnosis was 54 years (mean 53, range 12–82), and at surgery for BM the median age was 62 years (mean 59, range 12–82). Forty-three patients were identified that underwent surgery for bone metastases. Two patients with surgery for lesions of the spine or cranium were excluded as they were performed by Neurosurgeons. Forty one patients underwent operative intervention by the Musculoskeletal Oncology Service at 10 different sites for disease in the pelvis or appendicular skeleton. Patient demographics are summarized in Table 1. Twenty-five patients (61%) had synchronous bone metastases at the time of thyroid cancer diagnosis. Sixteen patients (39%) developed metachronous bone metastasis after thyroidectomy for the primary tumor.

**Figure 1:**
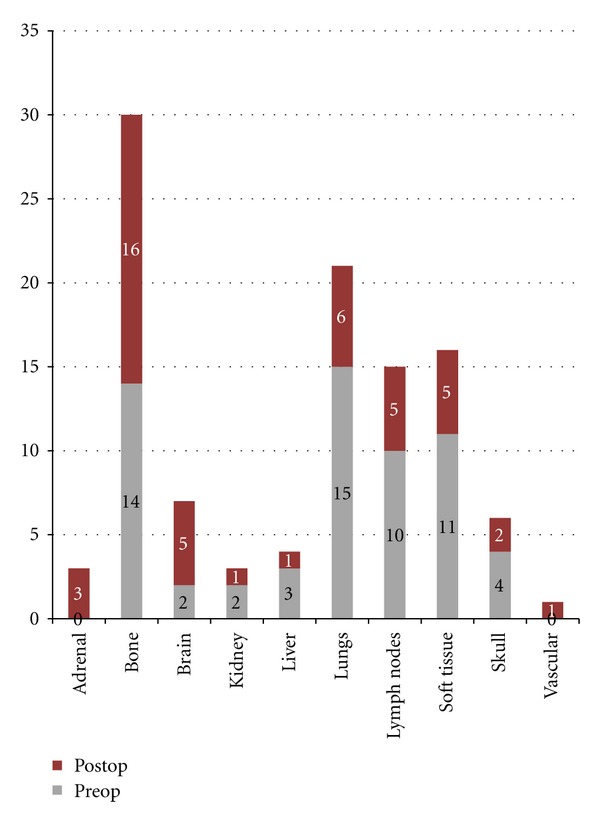
The distribution of other metastatic disease sites at the time of surgical intervention for osseous metastasis is shown in the lower bars. After surgery, the sites of subsequent metastases formation are shown in the upper bars. The most common site of metastatic disease prior to surgery was lung. Following surgery, bone was the most common site of new metastatic disease.

Twelve patients presented with solitary bone metastasis (only one bone involved). Six of 12 patients with single bone metastasis had no other metastastic distant organ involvement. Two of the patients with single bone involvement had lung metastasis with no other organ involvement. The remaining 4 patients with single bone involvement had synchronous metastases in the lymph nodes, liver, and soft tissue. The remaining 29 patients presented with metastases involving multiple bones. The most common site of synchronous metastasis in these patients was the lung (Figure 1). Bone, soft tissue, and lymph nodes were the most common locations for metachronous metastasis. 

Patients were followed for a minimum of 10 months unless they died prior to 12 months. The median duration of the followup was 60 months (10–102 range). At the time of the last followup, 11 of 41 patients were alive. All deaths were secondary to disease progression. Date of patient death was determined from patient records and the Social Security Death Index.

### 2.3. Surgery

The site and type of surgery are summarized in Table 2. The indications for surgery included diagnosis (biopsy), pathologic fracture, impending pathologic fracture, and intractable pain. Thirty-four patients had a surgery that included tumor removal (curettage or wide resection), and seven patients did not (biopsy and bone stabilizing procedures). The total number of patients presenting at our institution with bone metastasis is shown in Table 3. Of the 8681 patients presenting with thyroid carcinoma, 550 patients developed bone metastases, and 41 required surgery in the appendicular skeleton. For the 41 surgical cases, the histological diagnosis was follicular carcinoma for 21 (51%), papillary carcinoma for 6 (15%), Hurthle cell carcinoma for 10 (24%), medullary carcinoma for 2 (5%), and anaplastic for 2 (5%). The most common surgical site was the femur, followed by the humerus, pelvis, radius, and then scapula. 

Thirty-seven of the 41 surgical patients were treated with radioactive iodine (^131^I). At our institution, the standard treatment for patients that present without metastasis (Stage I disease) is thyroidectomy and lymph node dissection, followed by radioactive iodine for the initial treatment. Patients presenting with bone metastasis (or other distant organ involvement) also have thyroidectomy and then receive radioactive iodine if there is no prior history of treatment, and/or iodine tracer uptake at metastatic sites. 

Seven of the 41 surgical patients had preoperative radiation to the bone metastatic site. Four patients received chemotherapy instead of ^131^I. Thirty-eight of the patients had complete thyroidectomies (one patient had a partial thyroidectomy). Surgical treatment of bone metastases included excision in 34 cases (16 with en bloc resection and 18 with curettage). In 7 cases, the tumor was not removed, and the bone was simply stabilized. 

### 2.4. Statistical Analysis

Patient overall survival and local progression (recurrence) free survival was determined by Kaplan-Meier analysis, and the log rank test was used to compare the survival curves for different groups. Cox proportional hazards model were also fitted. Local progression was assessed by imaging studies. Local recurrence was defined as the reappearance on imaging studies of osseous of soft tissue tumor after prior excision. The Student's *t*-test was used to compare means. The association of blood loss volume with tumor excision was assessed using the Wilcoxon rank-sum test. SAS version 9.3 and S-Plus version 8.0 were used to perform all analyses. Statistical significance was defined as *P* < 0.05. 

## 3. Results

### 3.1. Survival

**Figure 2:**
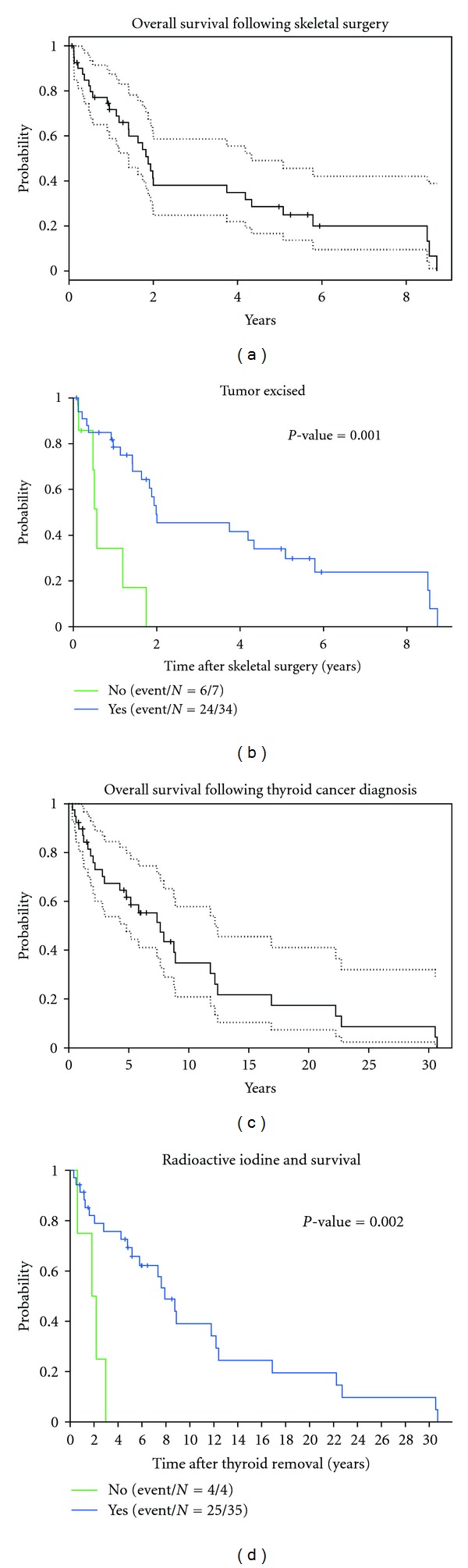
Kaplan-Meier analysis of patient survival. (a) Following surgery for osseous metastases patient survival was 72% at 1 year (95% CI 59–87%), 29% at 5 years (95% CI 17–49%), and 20% at 8 years (95% CI 10–42%). (b) There was better survival for patients when the metastastic osseous tumor was excised versus not excised. The median survival time for patients with tumor excision was 2 years (95% CI 1.4–5 years), compared with 0.6 years (95% CI 0.1–1.8 years) for patients without tumor excision (*P* = 0.001). No patient without tumor excision survived beyond 1.8 years. (c) Following thyroidectomy at initial diagnosis of thyroid carcinoma patient survival was 62% at 5 years (95% CI 48–80%), 35% at 10 years (95% CI 21–58%), 17% at 20 years (95% CI 7–41%), and 9% at 30 years (95% CI 2–32%). (d) After thyroid removal, patients who received radioactive iodine treatment had better survival than those who did not (*P* = 0.002).

Overall patient survival probability by Kaplan-Meier analysis after surgery for bone metastasis was 72% at 1 year, 29% at 5 years, and 20% at 8 years. Median survival from time of skeletal surgery was 1.9 years (range 1.2–4.2, Figure 2(a)). At the time of the last followup, 11 patients were alive. Patients who had their tumor excised had a lower risk of death following surgery adjusting for age and gender (*P* = 0.001, Figure 2(b)). Univariate analysis showed that radioactive iodine treatment, tumor subtype, chemotherapy, and preoperative radiation treatment had no effect on survival after skeletal surgery (Table 4). Patients with a solitary bone metastasis at the time of presentation also showed a trend for improved survival relative to those with multiple bone metastases, although not statistically significant (*P* = 0.07). Tumor excision and age were also significant prognostic factors by multivariate analysis (Table 5). 

In comparison, median survival after thyroidectomy in the same patient group was 5 years (range 0.8–8.5). Overall survival probability was 62% at 5 years and 35% at 10 years (Figure 2(c)). After thyroidectomy, patients treated with radioactive iodine had better survival than those who did not (*P* = 0.002, Figure 2(d)). 

**Figure 3:**
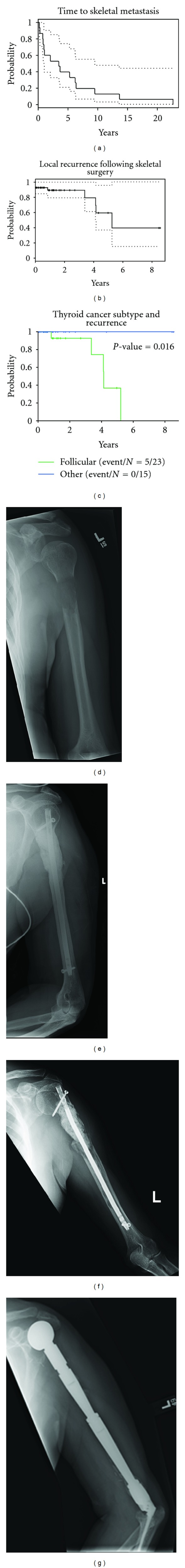
Progression of disease in bone. (a) Kaplan-Meier analysis of time to formation of skeletal metastasis. The median time to formation of bone metastasis after thyroid removal was 3.44 years. (b) Kaplan-Meier analysis of time to recurrence. The probability of recurrence free survival was 89% at 1 year (95% CI 80–100%), 60% at 5 years (95% CI 37–96%), and 40% at 8 years (95% CI 16–100%). (c) A diagnosis of follicular thyroid carcinoma was associated with a higher risk of recurrence versus other diagnoses (papillary, medullary, anaplastic, and Hurthle cell) (*P* = 0.016). All recurrences following surgery for osseous metastasis were in patients with follicular thyroid carcinoma histologic diagnosis. (d) A patient with an impending fracture of the proximal humerus shaft from isolated metastasis from follicular thyroid carcinoma underwent open nailing with curettage and cementation. (e) Postoperative X-ray demonstrates a humeral nail and bone cement filling the defect created by curettage of the metastasis. (f) Follow-up X-ray 5 years after surgery showing increasing lytic changes in bone extending to the distal aspect of the humerus, consistent with local progression of disease. (g) Postoperative X-ray showing total humerus endoprosthesis. The humerus, intramedullary nail, and bone cement were resected en bloc. The total humerus endoprosthesis reconstruction has remained stable, and the patient remains alive with no sign of disease.

The majority of patients (*n* = 23) had BM at the time of diagnosis of thyroid cancer. The median time to metastasis for patients (*n* = 16) without distant disease at the time of thyroid cancer diagnosis was 2.72 years (Figure 3(a)). In these patients the time to metastasis had no dependence thyroid tumor subtype or treatment modality. 

Serum thyroglobulin (TGB) levels were measured both pre- and postoperatively in 26 of 41 patients. In 22 patients, the serum TGB levels decreased following bone metastasis surgery. Preoperative serum TGBs ranged from 100 to 13000; with the percentage of decrease after bone metastasis surgery ranging from 9%–99%. In the 4 patients where there was no TGB decrease, 3 had widely metastasis disease involving multiple bones, and the skeletal surgery only addressed one site of bone involvement. The one remaining patient with no decrease in TGB had a biopsy, wherein the bone metastasis was not removed.

### 3.2. Local Recurrence

Eight of 41 cases were complicated by local recurrence. The local progression free survival was 89% at 1 year (80%–100%, 95% C.I.), 60% at 5 years (37%–96%, 95% C.I.), and 40% at 8 years (16%–100%, 95% C.I.) (Figure 3(b)). All cases of recurrence occurred in patients with a diagnosis of the follicular subtype of thyroid carcinoma, which was statistically significant via Kaplan-Meier analysis, in comparison to papillary, medullary, anaplastic, and Hurthle cell subtypes, where there were no recurrences (*P* = 0.016, Figure 3(c)). In contrast to overall survival, whether the metastasis was excised had no significant effect on the probability of local recurrence. All cases of local progression occurred in patients who had tumor excision. 

**Figure 4:**
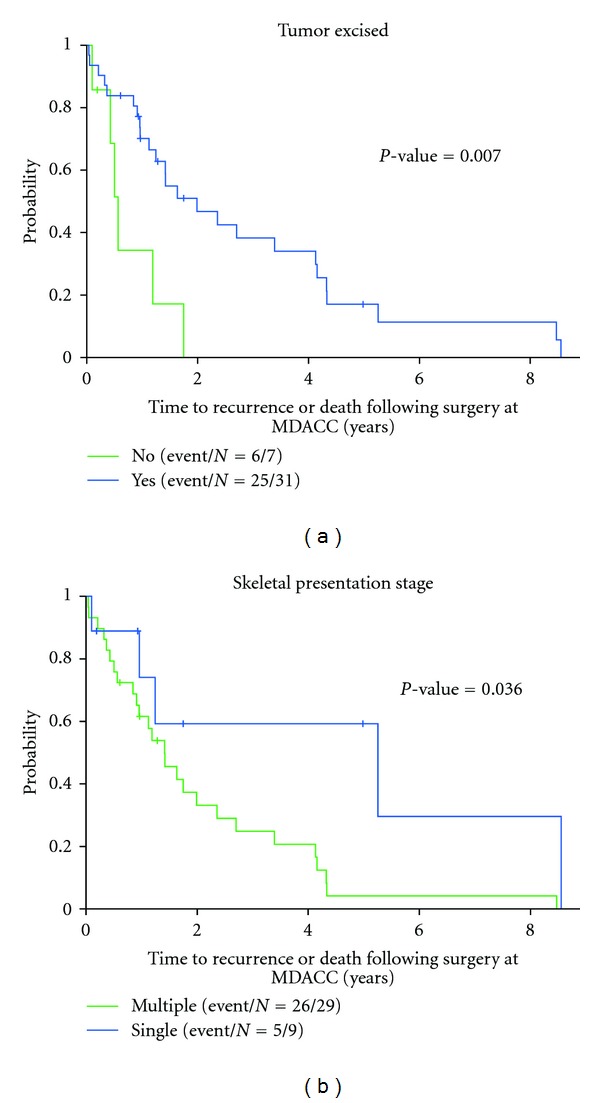
Kaplan-Meier analysis for recurrence free survival, as determined by the probability of no recurrence or death. (a) Patients had a higher probability of recurrence free survival if the osseous metastasis was excised versus no excision (*P* = 0.007). All patients that had surgeries where the metastasis were not excised died (*n* = 6) or were lost to followup (*n* = 1). (b) Patients had a higher probability of recurrence free survival if they presented with a single bone metastasis versus multiple bones (*P* = 0.036). Five of the 9 (56%) patients with single bone metastasis at presentation were alive at last followup, compared with 6 of 29 (21%) patients who presented with multiple bone metastasis.

In all cases of local progression, additional surgery was performed with hardware revision to either intercalary prosthesis, endoprosthesis, or joint replacement. One patient who was treated with an intramedullary nail for a humerus metastasis developed increasing pain, fracture, and progression of disease 4 years after surgery. The recurrent tumor was resected, and the nail was converted to a total humerus endoprosthesis (Figures 3(d)–3(g)). The patient was alive at the last followup 2 years after surgery to resect the recurrence. The hazard ratio for recurrence free survival was 0.28 (*P* = 0.012, 95% C.I. from 0.11 to 0.75, Table 4) for patients who had bone metastasis tumor excision compared to patients who did not. Kaplan-Meier analysis demonstrates worse recurrence free survival for patients who did not have surgical tumor excision for control of metastatic disease (Figure 4(a), *P* = 0.007), and for patients that presented with multiple sites of bone metastasis (Figure 4(b), *P* = 0.036). 

### 3.3. Radiation

**Figure 5:**
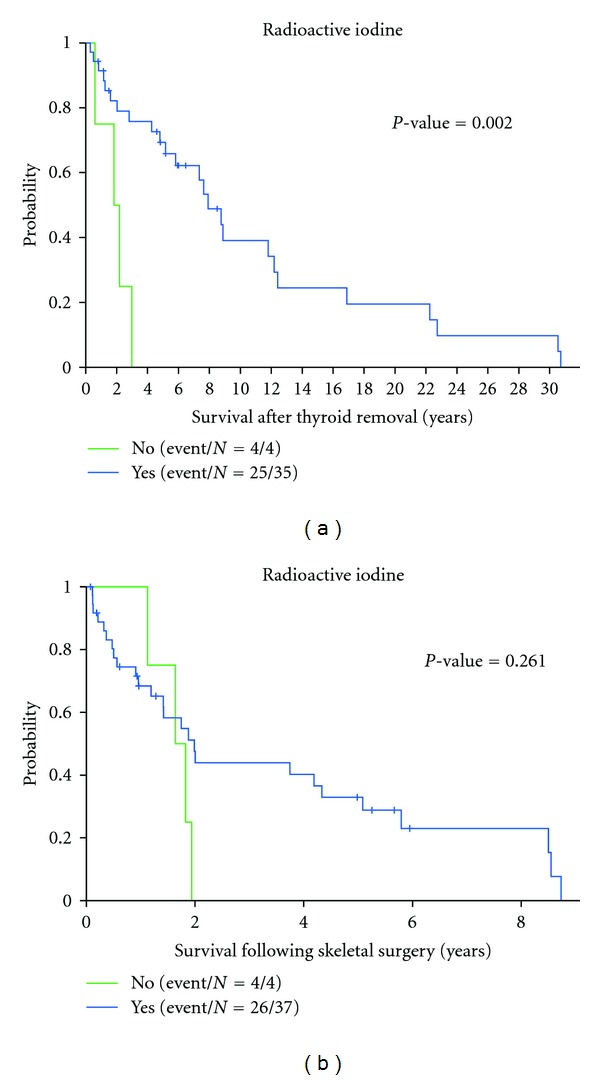
Kaplan-Meier analysis of the effect of radioactive iodine treatment on survival. (a) After thyroidectomy, patients treated with radioactive iodine had more favorable survival than those who were not candidates for radioactive iodine (*P* = 0.002). No patients who were not treated with radioactive iodine survived more than 3 years. (b) After surgery for osseous metastasis, there was no significant difference in survival between patients treated with radioactive iodine and those who were not (*P* = 0.261).

Patients treated with radioactive iodine (^131^I) had better survival following thyroidectomy (*P* = 0.002), but not following surgery for bone metastases (Figures 5(a)-5(b)). 

Seven patients failed prior to palliative external radiation of osseous metastases and subsequently underwent surgical treatment. Preoperative external beam radiation to the affected bone had no significant effect on overall patient survival after skeletal surgery (Table 4). There was a trend towards a higher risk of recurrence in patients who had preoperative radiation to the bone, although it did not achieve statistical significance (*P* = 0.08). One of the seven patients developed a local recurrence after surgery for the osseous lesion. This patient had excision of the recurrent lesion and hardware revision to acetabular reconstruction with total hip arthroplasty. 

### 3.4. Surgical Complications

Two patients had immediate perioperative complications. One patient with a history of smoking had respiratory insufficiency requiring delayed extubation. One other patient had atrial fibrillation. Both subsequently recovered uneventfully. There were no superficial or deep infections, nonunions, or perioperative deaths. 

The mean blood loss of 1014 mL (range 0–3900 mL) for the cases where the tumor was not removed (*n* = 7) did not differ significantly from cases where tumor was excised (mean = 1640 mL, range 0–19900 mL, *n* = 34). One patient became hemodynamically unstable during the second of bilateral procedures for skeletal metastases. Specifically, the patient became hypoxic and tachycardic during a hemiarthroplasty of the contralateral hip which immediately followed intramedullary nailing of the opposite femur. The hemiarthroplasty procedure was aborted, and the patient had an uneventful subsequent recovery. There were no deaths related to intraoperative blood loss.

## 4. Discussion

The results of this study suggest that metastatic thyroid cancer in bone is rare, but behaves aggressively. Surgery for bone metastasis in thyroid carcinoma has been infrequently studied [6–8]. In 23 years at our institution there were only 41 patients that required surgery for appendicular skeleton metastases. During the same period, 8681 patients with thyroid carcinoma were registered at our institution. Thus only approximately 0.5% of the patients required surgery for metastatic lesions in the appendicular skeleton. This study also shows that after bone metastasis occurs, survival is diminished, in agreement with published reports [1, 2, 7, 9, 10, 16]. Few patients survived more than 8 years after having surgery for bone metastasis [1, 15]. While it may seem appropriate that patients who have limited expected lifespan should receive conservative treatment, our study shows a survival advantage for patients who had undergone surgical excision of metastatic disease from bone. 

Thyroid cancer generally has an indolent nature, so that patients requiring skeletal surgery presented with bone metastasis both in late stages after treatment (16 of 41 patients) and with initial presentation (25 of 41 patients). Our study corroborates findings that survival with metastatic thyroid cancer tends to be better than some other cancers that metastasize to bone, such as renal cell carcinoma and lung carcinoma [2, 6, 14]. Three patients in this series survived for more than 30 years after their initial diagnosis, despite developing metastases in multiple bones and organs. The median survival for patients in our series was 7.6 years following initial diagnosis. 

Despite the survival advantage afforded by more aggressive tumor removal, whether a patient underwent tumor excision did not affect whether there was local progression for patients with follicular carcinoma subtype. There was, however, considerable morbidity for patients who developed uncontrolled tumor growth at metastatic osseous sites. In our study, all of the patients with local recurrence required additional surgical procedures. 

There was no survival advantage or disadvantage following thyroid cancer diagnosis or surgery for bone metastasis associated with subtype, although bone metastases are more common with the follicular subtype. Follicular thyroid cancer accounts for less than 15% of all differentiated thyroid cancers, but has a relatively high incidence of bone metastases, ranging from 7% to 28% [7, 9]. In our study, 6% of all patients developed bone metastases, and 7% of all patients had follicular carcinoma. Of the patients with follicular carcinoma, 15% developed bone metastases. Of the patients with bone metastases, 8% required skeletal surgery, and 21/43 (50%) of these patients had follicular carcinoma. All of the local recurrences occurred in patients with follicular thyroid carcinoma BM. Thus, the follicular subtype was associated with increased probability for local recurrence compared with other subtypes (Figure 3(c)). There was no selection bias for the surgical procedure used in patients with recurrence, as they were almost evenly divided between curettage (4) and wide resection (3). Therefore, the reasons for the aggressiveness of follicular thyroid carcinoma in bone are not identifiable from the limited patient cohort included in the current study but deserve further study. 

Bone metastases are much less common in papillary thyroid cancer, with a reported incidence from 1% to7% [14]. In our study, the incidence of papillary carcinoma was 3%. One comparable study that focused exclusively on patients with BM reported that in 96 patients, 17% had papillary histological diagnosis, and 71% were follicular [1]. Our study cohort had similar proportionality, with three times as many patients with BM from follicular thyroid cancer than papillary thyroid cancer. 

Anaplastic thyroid carcinoma typically has a poor prognosis. It is known to be the most lethal among all thyroid cancers, with median life expectancies reported to be from 4 to 12 months [30–34]. Our results are consistent with more recent reports of marginally improved survival with multimodal treatment strategies [31, 32]. Both patients with anaplastic thyroid carcinoma in our study received additional chemotherapy (doxorubicin based) and external radiation. One patient died at 23 months and the other died at 27 months following diagnosis. 

Studies show that more than 80% of bone metastases from all thyroid tumors are located in the axial skeleton [2]. Correspondingly, 41 out of 43 patients (95%) in our cohort had axial BMs. The propensity for the axial skeleton is thought to be secondary to blood flow distribution characteristics, and the bone microenvironment, which includes growth factors favorable for tumor growth [2].

There are few studies on survival following surgery for bone metastasis [3, 10, 35]. Reports have shown that removal of up to five bone metastases can be associated with improved survival and quality of life [1, 8, 15]. Therefore, some have strongly recommended surgical excision for accessible, solitary metastases. Our study results agree with this, especially for patients without extra-skeletal metastases. In these patients, surgical extirpation of solitary bone metastases was associated with improved survival. 

Total thyroidectomy followed by radioactive iodine is the treatment most often recommended for patients with synchronous distant metastases to bone. It has been argued that ^131^I is the only opportunity to slow progression and to prolong survival [5]. Our study shows that patients treated with radioactive iodine have a clear survival advantage following thyroidectomy, that is less clear and likely diminished following skeletal surgery (Figure 5). A possible explanation is that the patients undergoing skeletal surgery have developed bone metastases despite receiving ^131^I and therefore have more aggressive disease. A similar trend was observed in patients that failed palliative external radiation. The prognosis for these patients was worse, although it did not achieve statistical significance. Of the 7 patients that failed palliative XRT, 5 were known to have died within 4 years of surgery, with the other 2 patients lost to followup. 

The involvement of multiple bones precludes curative resection of bony disease. Of the 24 patients who had excision of the bone metastasis, 10 had solitary bone involvement. At the last followup 5 of the 10 were alive, 4 died, and one was lost to followup. Others studies have reported similar outcomes, with a significant improvement in survival with complete resection of skeletal metastases [1, 6]. For patients that presented with multiple synchronous bone metastases, surgical intervention afforded palliation for symptomatic disease that failed nonoperative interventions. Palliative surgery partially removes bone tumor or completely resects bone tumor but leaves residual tumor in other organs [7]. In accordance with this, there was poorer recurrence free survival observed in patients with multiple bone involvement. 

In patients with multiple organ involvement, the role of metastectomy is less well understood. In this study, two patients with solitary skeletal metastasis at presentation did not have surgical extirpation of the BM because of synchronous involvement in other organs. One of these patients had stabilization of a lesion in the radius with a plate, followed by external radiation. This patient was alive at 2-year followup, but went on to develop additional bone lesions in the clavicle and scapula. The other patient had synchronous lesions in the lungs and liver and a solitary lesion in the femur that was stabilized by intramedullary nail and treated with external radiation postoperatively. The patient died 8 months after surgery.

In one compendium of 13 studies, the rate of bone metastasis was found to be 25% among 1231 patients [12]. A more recent study showed that survival decreased by 14% in patients older than 40 years with lung metastases or multiple bone metastases compared with comparable cohorts [9]. Other studies suggest that patients older than 45 years have a poorer prognosis for remission following treatment with radioactive iodine [2]. Our findings are consistent with these reports. 

## 5. Conclusions

In conclusion, the development of osseous metastases from thyroid carcinoma is associated with poorer patient survival and prognosis. Patients with multiple bone metastases had poorer survival than those with solitary bone metastases. While 28% of patients died within a year of surgery for bone metastases, 20% remained alive at 8 years. Patients who responded to radioactive iodine had the best long-term survival. Multivariate analysis showed that both presentation with a single BM and complete BM resection surgery were significantly associated with improved patient and implant survival following surgery for skeletal metastases. Follicular thyroid cancer is the most common type associated with bone metastasis and recurs locally more often after surgical treatment. More work will be needed to fully understand and identify the factors that determine which patients survive longer and have greater need for local control. Thus, it remains necessary to allow for the surgeon's judgment as to which patient should undergo tumor excision as a part of the metastatic tumor treatment. 

## Figures and Tables

**Table 1:** Patient demographics.

Characteristic	Number (total *n* = 41)
Gender	
Female	22
Male	19
Age (years)	
Mean	59
Range	12–82
Followup (months)	
Median	60
Range	10–102
Surgery sites	
*Upper extremity *(*n* = 16)	
Scapula	1
Proximal humerus	5
Shaft humerus	4
Distal humerus	3
Radius	2
Metacarpal	1
*Lower extremity *(*n* = 25)	
Acetabulum	8
Proximal femur	12
Shaft femur	4
Distal femur	1
Skeletal presentation stage	
Solitary bone metastasis	12
Multiple bone metastases	29

**Table 2:** Types of surgery.

Tumor removal	Category	Procedure	Number (*n* = 41)
No (7 cases)	EBL	Median	150 cc
		Range	0–2500 cc
		Nail (closed)	3
		Plate	1
		Biopsy	3

Yes (34 cases)	EBL	Median	775 cc
		Range	0–19900 cc
	Curettage (19 cases)	Nail + PMMA	9
		Plate + PMMA	1
		Pins + PMMA	4
		THA	5
	Wide resection (15 cases)	Endoprosthesis	12
		No reconstruction	3

**Table 3:** Thyroid cancer diagnosis subtypes, bone metastases, and bone surgery sites.

Thyroid cancer subtype	Total number of patients	Patients with bone metastases (percentage of total)	Patients who had surgery on bone metastases (percentage of pts with bone mets)	Surgery sites	Other treatments (patients treated/total getting surgery)
*Anaplastic *	264	22 (8.3)	2 (9.1)	Femur (2)	XRT, chemo (2/2)
*Follicular *	637	96 (15.1)	21 (21.9)	Hand metacarpal (1), radius (1), humerus (6), pelvis (5), femur (8)	XRT (2/21)
*Hurthle *	342	44 (12.9)	10 (22.7)	Humerus (5), scapula (1), pelvis (2), femur (2)	XRT, chemo (4/10)
*Medullary *	866	134 (15.5)	2 (1.5)	Humerus (1), femur (1)	Chemo (1/2)
*Papillary *	6572	254 (3.9)	6 (2.4)	Radius (1), humerus (1), femur (4)	None

*Total*	8681	550 (6.3)	41 (7.5)		

**Table 4:** Univariate Cox model.

Prognostic factors	Overall survival			Time to bone metastasis			Survival after bone metastasis surgery			Recurrence free survival		
	Hazard ratio	95% CI	*P* value	Hazard ratio	95% CI	*P* value	Hazard ratio	95% CI	*P* value	Hazard ratio	95% CI	*P* value
Age	1.06	1.02–1.09	**0.001**	1.06	1.005–1.116	**0.032**	1.002	0.975–1.03	0.864	1.012	0.98–1.04	0.41
Gender F v M	1.19	0.55–2.38	0.66	0.63	0.21–1.83	0.39	1.63	0.76–3.50	0.21	1.795	0.87–3.7	0.113
Follicular subtype	1.1	0.51–2.37	0.81	1.26	0.43–3.71	0.68	0.83	0.40–1.73	0.63	1.33	0.63–2.81	0.45
Radioactive iodine Y v N	0.18	0.05–0.61	**0.006**	0.07	0.005–1.186	0.066	0.54	0.18–1.62	0.27	0.504	0.16–1.53	0.23
Other treatment Y v N	1.51	0.63–3.62	0.36	1.721	0.55–5.34	0.35	0.95	0.40–2.25	0.91	0.72	0.29–1.78	0.48
Skeletal presentation Multiple bone v single							2.44	0.93–6.44	0.07	3.03	1.03–8.92	**0.044**
Preoperative radiation to bone Y v N							1.985	0.73–5.38	0.18	1.74	0.65–4.68	0.27
Tumor excised from bone Y v N							0.21	0.075–0.58	**0.003**	0.28	0.11–0.75	**0.012**

**Table 5:** Multivariate Cox model.

Prognostic factors	Overall survival			Survival after bone metastasis surgery			Recurrence free survival		
	Hazard ratio	95% CI	*P* value	Hazard ratio	95% CI	*P* value	Hazard ratio	95% CI	*P* value
Age	1.054	1.02–1.09	**0.002**						
Radioactive iodine Y v N	0.164	0.041–0.654	**0.0104**						
Follicular subtype	1.589	0.646–3.910	0.313	0.832	0.396–1.748	0.627	1.547	0.706–3.39	0.276
Skeletal presentation Multiple bone v single				2.102	0.783–5.643	0.141	3.097	1.008–9.521	**0.0485**
Tumor excised from bone				0.242	0.087–0.677	**0.0068**	0.336	0.125–0.903	**0.0306**
